# Biogeophysical Impacts of Land‐Use Change on Climate Extremes in Low‐Emission Scenarios: Results From HAPPI‐Land

**DOI:** 10.1002/2017EF000744

**Published:** 2018-03-08

**Authors:** Annette L. Hirsch, Benoit P. Guillod, Sonia I. Seneviratne, Urs Beyerle, Lena R. Boysen, Victor Brovkin, Edouard L. Davin, Jonathan C. Doelman, Hyungjun Kim, Daniel M. Mitchell, Tomoko Nitta, Hideo Shiogama, Sarah Sparrow, Elke Stehfest, Detlef P. van Vuuren, Simon Wilson

**Affiliations:** ^1^ Institute for Atmospheric and Climate Science Eidgenössische Technische Hochschule (ETH) Zurich Zurich Switzerland; ^2^ Institute for Environmental Decisions Eidgenössische Technische Hochschule (ETH) Zurich Zurich Switzerland; ^3^ Land in the Earth System, Max Planck Institute for Meteorology Hamburg Germany; ^4^ PBL Netherlands Environmental Assessment Agency Den Haag The Netherlands; ^5^ Institute of Industrial Science The University of Tokyo Tokyo Japan; ^6^ School of Geographical Sciences University of Bristol Bristol UK; ^7^ Center for Global Environmental Research National Institute for Environmental Studies Tsukuba Japan; ^8^ Oxford e‐Research Centre (OeRC) University of Oxford Oxford UK; ^9^ Copernicus Institute for Sustainable Development Utrecht University Utrecht The Netherlands; ^10^ Met Office Hadley Centre Exeter UK; ^11^ Department of Meteorology, NCAS‐CMS University of Reading Reading UK

**Keywords:** HAPPI‐Land, land‐use change, land‐based mitigation, Shared Socioeconomic Pathways, Earth System Modeling, Integrated Assessment Modeling‐

## Abstract

The impacts of land use have been shown to have considerable influence on regional climate. With the recent international commitment to limit global warming to well below 2°C, emission reductions need to be ambitious and could involve major land‐use change (LUC). Land‐based mitigation efforts to curb emissions growth include increasing terrestrial carbon sequestration through reforestation, or the adoption of bioenergy crops. These activities influence local climate through biogeophysical feedbacks, however, it is uncertain how important they are for a 1.5° climate target. This was the motivation for HAPPI‐Land: the half a degree additional warming, prognosis, and projected impacts—land‐use scenario experiment. Using four Earth system models, we present the first multimodel results from HAPPI‐Land and demonstrate the critical role of land use for understanding the characteristics of regional climate extremes in low‐emission scenarios. In particular, our results show that changes in temperature extremes due to LUC are comparable in magnitude to changes arising from half a degree of global warming. We also demonstrate that LUC contributes to more than 20% of the change in temperature extremes for large land areas concentrated over the Northern Hemisphere. However, we also identify sources of uncertainty that influence the multimodel consensus of our results including how LUC is implemented and the corresponding biogeophysical feedbacks that perturb climate. Therefore, our results highlight the urgent need to resolve the challenges in implementing LUC across models to quantify the impacts and consider how LUC contributes to regional changes in extremes associated with sustainable development pathways.

## Key Points

- Land‐use change (LUC) accounts for 20% change in temperature extremes for low‐emission scenarios
- Multimodel results show projected temperature extremes depend on where and what land‐based mitigation activities are pursued
- For some regions and models, LUC can affect temperature extremes as much as a half degree change in global mean temperature

## 1 Introduction

The impacts of land use and land management for food production and forestry have been shown to have considerable influence on local and regional climate, particularly for climate extremes (Avila et al., 2012; Davin et al., 2014; de Noblet‐Ducourdé et al., 2012; Hirsch et al., 2015, 2017; Lejeune et al., 2017; Luyssaert et al., 2014; Pielke et al., 2011; Pitman et al., 2009, 2012). With the recent commitment to limit global warming to well below 2°C (United Nations Framework Convention on Climate Change [UNFCCC], 2015) and to pursue efforts to stabilize at 1.5°C, emission reductions need to be ambitious and could involve major land‐based mitigation measures (Popp et al., 2017; van Vuuren et al., 2011). These measures could lead to substantial land‐use change (LUC), such as increasing terrestrial carbon sequestration through reforestation, increasing bioenergy plantations, and adapting agricultural management to promote carbon sequestration in soils (e.g., residue management, biochar, and reduced tillage). All of these activities also have an impact on local and regional climate through biogeophysical feedbacks associated with changes in albedo, roughness, and evapotranspiration that accompany LUC (Bonan, 2008; Bright et al., 2017; Jones et al., 2013; Lejeune et al., 2017; Winckler et al., 2017). This is expected to be particularly important in the transitional regions between wet and dry climates, where land conditions have been shown to substantially affect projected changes in climate variability and extremes (Seneviratne et al., 2010, 2016). However, there is considerable uncertainty on how important these feedbacks are for half a degree of warming.

This article presents results from the HAPPI‐Land climate model intercomparison project (half a degree additional warming, prognosis, and projected impacts—land‐use scenario experiment) that aims to assess LUC‐based uncertainties in low‐emission scenarios. HAPPI‐Land constitutes an extension to the existing HAPPI multimodel experiment (Mitchell et al., 2016, 2017) that assesses changes in climate for half a degree additional warming. In HAPPI‐Land, the experimental setup is expanded to consider the impacts of LUC in a multimodel framework.

There are several reasons why we need to understand how land‐based mitigation activities may influence climate. First, the basis for climate projections from Earth system models (ESMs) relies upon scenarios created from integrated assessment models (IAMs), which includes providing spatially explicit information on land use associated with land‐based mitigation (e.g., van Vuuren et al., 2011). Second, these scenarios rely upon assumptions about future developments that are difficult to predict, particularly in the context of socioeconomic factors including population growth and fossil fuel dependency. Finally, climate change may impose constraints on where land‐based mitigation is feasible in terms of water availability and competition for land (Boysen et al., 2017; Smith et al., 2010). Critically, information on the biogeophysical feedbacks of LUC on climate are currently not considered by IAMs (Jones et al., 2013; Seneviratne et al., 2018), in part due to the uncertainty on what these feedbacks are and the uncertainties arising from how the future may evolve. Therefore, we need to examine how the implementation of land‐use patterns of IAM scenarios contributes to climate impacts in ESMs. In this study, we focus on the uncertainties arising from LUC.

Historically, LUC at a global scale has involved extensive conversion of forests into pasture and croplands to support food production (Hurtt et al., 2006). While the biogeochemical impacts of deforestation are known to increase radiative forcing from increased CO_2_ (Boysen et al., 2014; Brovkin et al., 2013; Sitch et al., 2005), the biogeophysical impacts from historical LUC are less certain. Forest to cropland transitions generally result in increased surface albedo that has the potential to cool the surface locally by decreasing the amount of radiation absorbed by the land surface. However, LUC also changes the partitioning between the surface turbulent energy fluxes, and subsequently the cooling or warming resulting from this conversion depends on where the deforestation occurs (Alkama & Cescatti, 2016; Bonan, 2008; Bright et al., 2017). This has presented a considerable challenge to the ESM community with previous multimodel LUC studies (e.g., Land‐Use and Climate, IDentification of robust impacts [LUCID] and Coupled Model Intercomparison Project [CMIP5]) showing contrasting impacts on temperature that are even greater for extremes (Boisier et al., 2012; de Noblet‐Ducoudré et al., 2012; Lejeune et al., 2017; Pitman et al., 2009). This is likely a result of differences in how albedo and evapotranspiration change in response to LUC (Lejeune et al., 2017). Therefore, the competing influences of albedo and evapotranspiration change on surface temperature contribute to model uncertainty on how LUC has influenced past climate which is also highly relevant for how LUC arising from climate change mitigation may influence future climate.

In this study, we present the first multimodel results from HAPPI‐Land to evaluate the uncertainty in predicting extremes for half a degree warming arising from two distinct land‐use scenarios compatible with low‐emission scenarios. This includes one scenario that prioritizes reforestation and another that involves widespread adoption of bioenergy crops. We further aim to explain why modeled differences in climate extremes arise from the biogeophysical feedbacks of LUC through changes in the surface energy balance (SEB).

## 2 Materials and Methods

### 2.1 Land‐Use Scenarios From IAM

**Figure 1:**
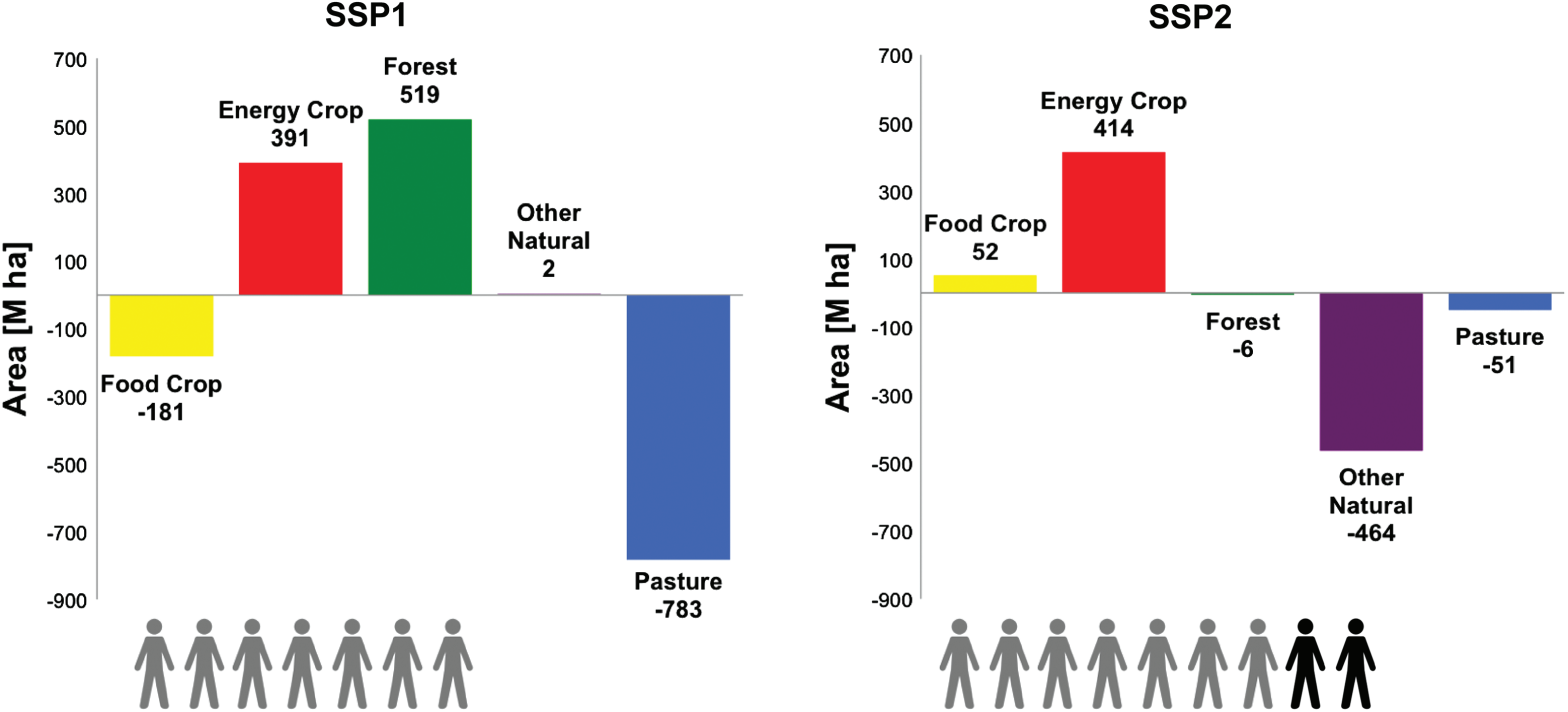
Summary schematic of major land conversions applied in SSP1‐1.9 (left) and SSP2‐1.9 (right) between 2100 and 2010. Land area converted in units of M ha. The person icons denote the global population with one icon representative of 1 billion people.

New scenarios have been developed for the sixth phase of the CMIP6 that are combined with the existing representative concentration pathways (RCPs) from CMIP5 to include socioeconomic assumptions based on the shared socioeconomic pathways (SSPs) (Riahi et al., 2017). The land‐use scenarios examined in HAPPI‐Land were developed using the Integrated Model to Assess the Global Environment (IMAGE) version 3.0 (Stehfest et al., 2014). The IMAGE model is a simulation model that comprises various submodels describing the energy system, the agricultural economy, land use, natural vegetation, hydrology, the climate system, and climate change mitigation policy. Most of the socioeconomic variables are modeled for 26 regions, while most of the environmental processes are modeled on a latitude/longitude grid (5″ or 30″ resolution). The scenarios used in this study were developed according to various qualitative and quantitative assumptions as agreed upon in a collaborative process in the climate change research community (Riahi et al., 2017). Each baseline SSP scenario is combined with various climate change mitigation targets consistent with the RCPs (van Vuuren et al., 2011). To achieve a certain climate target, carbon tax trajectories and scenario‐specific assumptions are used to derive a range of mitigation measures affecting the energy, industry and agricultural sectors, and land use. The latter, through the large area used for bioenergy and afforestation, is of particular relevance to this study. Therefore, the land‐use scenarios used in HAPPI‐Land correspond to the low‐emission scenario RCP1.9, which is consistent with a 1.5°C global warming target under two SSPs: the SSP1 “green growth strategy” and the SSP2 “middle‐of‐the‐road development pattern” (Doelman et al., 2018; van Vuuren et al., 2017). The SSP1 scenario involves strong reforestation at the expense of grasslands due to limited growth in demand for animal products and large efficiency gains in livestock production systems. The SSP2 scenario involves an ongoing expansion of croplands and livestock production (consistent with the historical trend) contributing to a decrease in forest area in some regions. However, the climate policies in the SSP2 mitigation case lead to a small increase in forest area in different regions due to the protection of forest carbon stocks. The main differences in land cover between the SSP‐1.9 land‐use scenarios (compatible with the 1.5°C climate warming target) are depicted in Figure 1. Here, we note that we also examined the IMAGE land‐use scenarios for a 2.6 W m^−2^ target (i.e. RCP2.6) under the two SSPs and found them to have similar land‐cover distributions despite differences in radiative forcing and CO_2_ concentrations (see Figure S1, Supporting Information). Therefore, we only use the land‐use scenario from IMAGE corresponding to SSP1‐1.9 and SSP2‐1.9, as these are also representative of the major features of SSP1‐2.6 and SSP2‐2.6, respectively (see Doelman et al., 2018 for more details on the IMAGE scenarios). Hereafter, we use SSP1 and SSP2 to refer to the land‐use scenarios under RCP1.9.

### 2.2 Climate Models and Experimental Protocol

Four climate models have been used to perform a common set of simulations for HAPPI‐Land. This includes the Community Earth System Model (CESM), the Hadley Center Atmospheric general circulation Model 3P (HadAM3P), the Model for Interdisciplinary Research On Climate (MIROC), and the Max Planck Institute Earth System Model (MPI‐ESM). These models, including version and resolution information, are listed in Table 1 along with the key reference material.

**Table 1:** Climate Models Contributing to HAPPI‐Land

Model	Version	Resolution (longitute × latitude)	Model years per experiment	References
CESM	CAM4 CLM4CN	2° × 2°	200	Neale et al. (2013)
HadAM3P	2 MOSES2	1.88° × 1.25°	300	Guillod et al. (2017)
MIROC	5	150 × 150 km	200	Shiogama et al. (2014)
MPI‐ESM	ECHAM6.3	1.9° × 1.9° (T63)	100	Giorgetta et al. (2013)

The experimental design follows the HAPPI protocol (Mitchell et al., 2017), which consists of coupled land–atmosphere initial condition ensemble simulations with prescribed sea surface temperatures (SSTs), sea ice, greenhouse gas (GHG) and aerosol concentrations, and solar and volcanic activity that coincide with three forced climate states. This includes a present climate decade (2006–2015), which employs observed external forcings (such as observed SSTs, sea ice, GHG, and land use) and two future climate decades (2091–2100) with the global mean temperature forced to 1.5°C (Plus15) or 2.0°C (Plus20). The Plus15 future climate uses the atmospheric GHG and aerosol concentrations of the year 2095 from RCP2.6. RCP2.6 is used because coincidently it stabilizes at 1.5°C at the end of the century in terms of CMIP5 multimodel mean (Mitchell et al., 2017). For the SSTs and sea ice, the CMIP5 multimodel mean SST/sea‐ice anomaly for 2091–2100 relative to present day (PD) is added to the observed values, which were used in the current decade experiment. The Plus20 future scenario employs a similar experimental design, but uses a weighted combination of RCP2.6 and RCP4.5 multimodel mean for the GHGs, SSTs, and sea ice, as there is no one RCP scenario that directly stabilizes at 2°C. As aerosols are not well mixed, and should not be averaged between two RCP scenarios, these are kept the same for the Plus20 scenario as with the Plus15 (see Mitchell et al., 2017 for details). For both Plus15 and Plus20, natural forcings (solar and volcanic activity) are the same as in the PD experiment. Given that this is also the case for SST and sea‐ice anomalies, the future scenarios can be seen as a replication of the PD decade in two future climate states.

In HAPPI, the land‐use scenario is the same in Plus15 and Plus20 and corresponds to LUC derived from RCP2.6. HAPPI‐Land expands on this by using three different land‐use scenarios for the Plus15 and Plus20 experiments. This includes one with no LUC by using PD land cover for the two future climate decades (Plus15_Hist_ and Plus20_Hist_). The other two land‐use scenarios correspond to the year 2095 from the SSP1 (Plus15_SSP1_ and Plus20_SSP1_) and SSP2 (Plus15_SSP2_ and Plus20_SSP2_), respectively. Therefore, land use is fixed for each future climate decade experiment. Note that Plus15_Hist_ and Plus20_Hist_ facilitate the evaluation of the effects of anthropogenic GHG warming in the absence of any LUC. Therefore, the only difference between the HAPPI and HAPPI‐Land Plus15 and Plus20 experiments is the land‐use scenario (which is fixed during the simulation); all other forcings are the same to avoid introducing any confounding factors to the results. All experiments analyzed in this manuscript are summarized in Table 2. The procedure for mapping of the SSP land‐use scenarios to the respective ESM plant functional types (PFTs) is also provided in the following section.

**Table 2:** Summary of All Experiments Performed for HAPPI‐Land

Project	Ensemble name	Climate forcing^a^	Land use
HAPPI^b^	Hist	Hist	Hist
HAPPI‐LAND	Plus15_Hist_	Plus15	Hist
	Plus20_Hist_	Plus20	Hist
	Plus15_SSP1_	Plus15	SSP1^c^
	Plus20_SSP1_	Plus20	SSP1^c^
	Plus15_SSP2_	Plus15	SSP2^c^
	Plus20_SSP2_	Plus20	SSP2^c^

a Including CO_2_, SST, and sea ice.

b CO_2_ concentration for the Hist ensemble 378–404 ppm.

c From the year 2095, and land use from the SSP1‐1.9 and SSP2‐1.9.

We follow the same approach as that employed in HAPPI (see Mitchell et al., 2017 for details). Here, each ensemble member of a given climate decade consists of a continuous 11‐year simulation where the last 10 years are used in the analysis assuming a 1‐year atmospheric spin‐up for all climate states. Ensemble generation method was left to the discretion of participants and a minimum of 10 ensemble members per experiment was requested from participants to enable a sufficient sample size for evaluating climate extremes.

### 2.3 Mapping of Land‐Use Scenarios to Model Plant Function Types

All participating ESMs have differences in how vegetation is described for the land surface particularly for the discretization of vegetation cover into PFTs. This includes how vegetation is prescribed, the number of PFTs, and whether pasture or crops are included as separate PFTs. These characteristics are provided in Table 3.

**Table 3:** Vegetation Characteristics of Climate Models Participating in HAPPI‐Land

Model	Land surface model	Land cover	No. PFTs	Tiling	Pasture	Crops
CESM (CAM4)	CLM4CN	Prescribed	16	Yes	No	Yes
HadAM3P	MOSES2	Prescribed	9	Yes	No	No^a^
MIROC5	MATSIRO	Prescribed	11	Partial^b^	No	Yes
MPI‐ESM (ECHAM6.3)	JSBACH	Prescribed	11	Yes	Yes	Yes

a Crop changes in the SSPs are allocated to the C3 grass PFT while changes in pasture are allocated to the C4 grass PFT.

b Each grid cell comprises a cropland tile and a natural vegetation tile of the dominant type among 10 potential PFTs.

To minimize potential inconsistencies, we recommended the following procedure to map the IMAGE land use to the PFTs of the participating models. First, we conservatively aggregated the IMAGE land use to the model resolution. Then, we apply the change (2095 minus 2010) in the IMAGE land use (for crops, pasture, forest, and other naturals) to the 2010 land cover used in the present climate ensemble. For CESM changes in pasture from the IMAGE, SSPs were applied to the grass PFT of that model. For HadAM3P, which does not have a separate crop PFT, IMAGE crop changes were allocated to the C3 grass PFT and pasture changes allocated to the C4 grass PFT. MIROC cannot explicitly consider pasture changes as only one natural (or potential) vegetation PFT is allowed per grid cell. Therefore, the MIROC simulations only consider changes in the fractional cropland area. The natural vegetation distribution in MPI‐ESM is based on a land‐cover reconstruction by Pongratz et al. (2008) to which crop and pasture changes from IMAGE are added. A special feature of MPI‐ESM is that pasture expansion first happens on natural grassland before other natural vegetation like shrubs or forests are removed. The implementation of the IMAGE land‐use scenarios is presented in Section 3.

### 2.4 Analysis

Our analysis uses the daily output from each model to examine changes in the extremes indices. These indices include the annual maximum daytime 2 m air temperature (TXx) and the annual minimum night‐time 2 m air temperature (TNn, analyses in Supporting Information). To test whether the change in the extreme indices is statistically significant between the ensemble means of two experiments, we generate a 1000 bootstrap sample distribution of the paired differences, where we sample with replacement. The change in the extreme indices is statistically significant if the 95% confidence interval of the 1000 bootstrapped differences does not include zero (i.e., the null hypothesis that the two ensemble means do not differ is rejected).

For a given diagnostic, we evaluate the impact of LUC in the context of all forcings (e.g., increased GHGs and SSTs) between the PD and the future climate states as

(1) $$\mathrm{LUC}\;\text{EFFECT}=|\frac{{\text{PlusZZ}}_{\mathrm{LU}}-{\text{PlusZZ}}_{\text{Hist}}}{{\text{PlusZZ}}_{\mathrm{LU}}-\text{Hist}}|$$

where ZZ corresponds to either the 1.5°C or 2.0°C climate state, LU is the land‐use scenario (i.e., SSP1 or SSP2), and Hist is the diagnostic value taken from the present climate ensemble. Therefore, the LUC EFFECT can be interpreted as the contribution of land use to the total change between Hist and PlusZZ_LU_.

Recognizing that not all models may respond in the same way to a comparable LUC forcing (Lorenz et al., 2016), we also examine changes in surface temperature (T_s_) in response to LUC using the SEB decomposition method (Hirsch et al., 2015; Luyssaert et al., 2014; Thiery et al., 2017; Vanden Broucke et al., 2015). Here, we express the SEB as

(2) $$\epsilon \sigma T_{s}^{4}=\left(1-\alpha \right){\mathrm{SW}}_{d}+{\mathrm{LW}}_{d}-Q_{E}-Q_{H}-R$$

where ϵ is the surface emissivity (we assume a constant value of 0.97 for all models), σ is the Stefan–Boltzmann constant (5.67 × 10^−8^ W m^−2^ K^−4^), α is the surface albedo, SW_d_ is the incoming shortwave radiation, LW_d_ is the incoming longwave radiation, Q_E_ is the latent heat flux, and Q_H_ is the sensible heat flux. The residual term (R) includes the ground heat flux and changes in subsurface heat storage. The change between the experiment and control (denoted as Δ) is calculated by taking the derivative of equation (2) with respect to T_s_ and solving for ΔT_s_

(3) $$\Delta T_{s}=\frac{1}{4\epsilon \sigma T_{s,\mathrm{CTL}}^{3}}\left(-{\mathrm{SW}}_{d}\Delta \alpha +\left(1-\alpha \right)\Delta {\mathrm{SW}}_{d}+\Delta {\mathrm{LW}}_{d}-\Delta Q_{E}-\Delta Q_{H}-\Delta R\right)$$

The residual term is calculated directly from the model outputs as

(4) $$R={\mathrm{SW}}_{u}-{\mathrm{SW}}_{d}+{\mathrm{LW}}_{u}-{\mathrm{LW}}_{d}-Q_{E}-Q_{H}$$

where SW_u_ and LW_u_ are the outgoing shortwave and longwave radiation fluxes, respectively.

To examine regional changes, we focus our analysis over regions where the land‐use transitions are extensive. This includes North America (NAM; 20–60 N, 105–60 W), South America (SAM; 35°S–0°N, 66–34 W), Europe (EUR; 30–60 N, 10 W–60°E), Tropical Africa (TAF; 5–50 N, 20 W–51°E), and South East Asia including China and India (SEA; 12°S–15°N, 60–145°E).

## 3 Results

### 3.1 Implementation of IAM Land‐Use Scenarios Into ESMs

**Figure 2:**
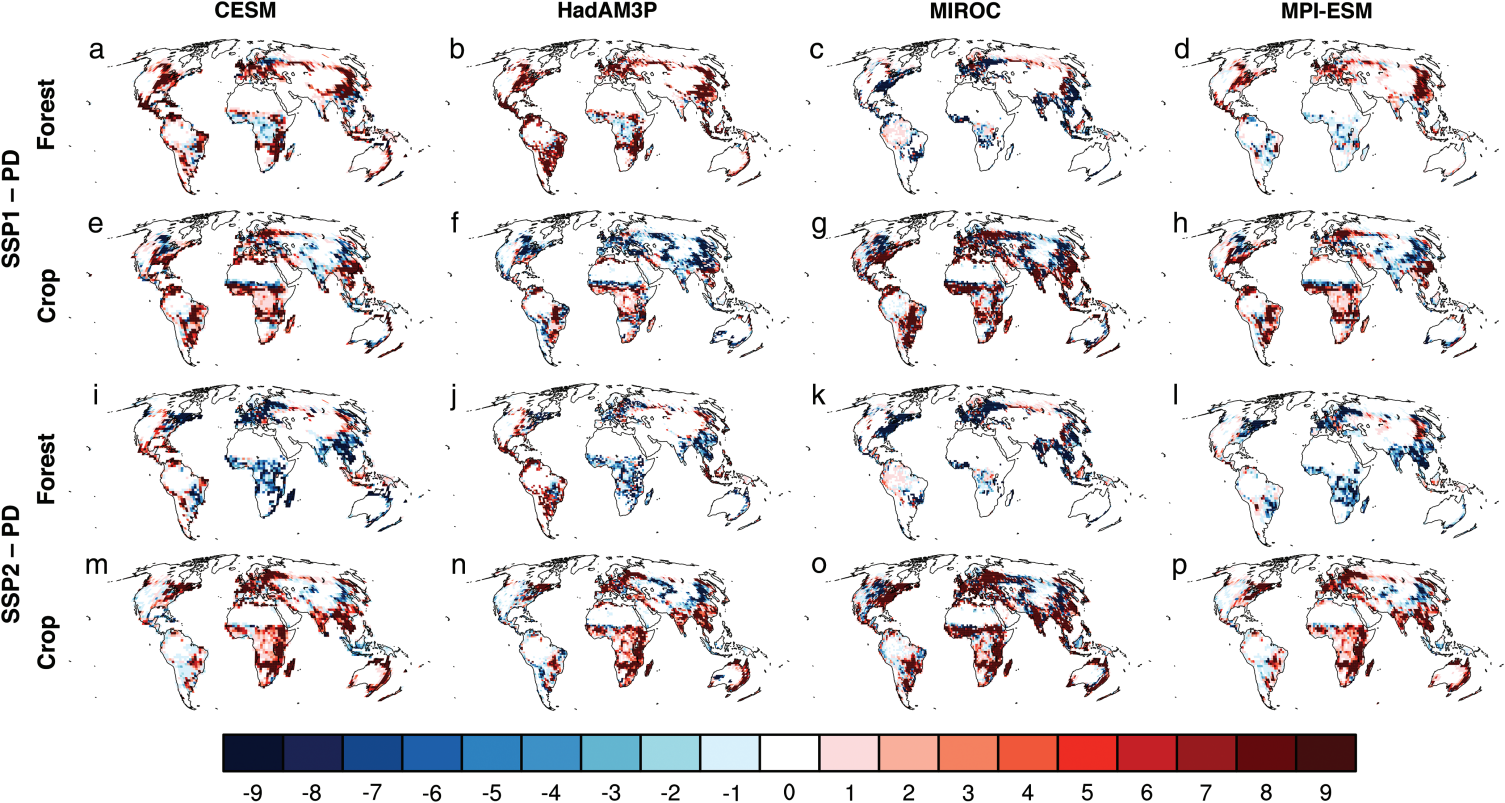
Crop and forest change as implemented in each participating model. Percent change in forest and crop PFTs for each participating model in HAPPI‐Land between year 2095 SSP land cover and year 2010 PD land cover. For CESM (a, e, i, and m), HadAM3P (b, f, j, and n), MIROC (c, g, k, and o), and MPI‐ESM (d, h, l, and p). Changes in SSP1 forests (a–d), SSP1 crops (e–h), SSP2 forests (i–l), and SSP2 crops (m–p).

As with previous multimodel LUC experiments (i.e., LUCID and CMIP5), a substantial challenge for HAPPI‐Land was maintaining a consistent implementation of the land‐use scenarios. We provided a set of guidelines for mapping the IAM land‐use scenarios to each participating group to minimize differences in the LUC forcing. Figure 2 illustrates the change in forest and crop PFTs relative to the present climate decade extents for the SSP1 and SSP2 land‐use scenarios as implemented in the participating ESMs. Although the general large‐scale features are similar across the models, with common regions where either deforestation or crop expansion occur, there are subtle differences in how large the percentage change is and in the spatial extent. For example, the MIROC model shows larger crop expansion in both land‐use scenarios (Figures 2g and 2o) compared to the other models. This may be associated with the tiling methodology applied in this model, which splits the grid cell between a crop fraction and a dominant vegetation type. Thus, the change in forest extents (Figures 2c and 2k) differs as well from the other models. Other examples include the reforestation over Africa in SSP1 in CESM and HadAM3P (Figures 2a and 2b), which is much larger in magnitude and spatial extent than that of MIROC and MPI‐ESM (Figures 2c and 2d). The net change in the main PFT classes for each ESM is listed in Table 4 including the initial area allocated to the main PFT classes. Again, it becomes evident that intermodel differences could not be entirely avoided. This is associated with differences in the PFT classification, particularly on how crops, grass, shrubs, and pasture have been defined and allocated in the respective models, the treatment of IMAGE pasture changes (as a pasture PFT in the MPI‐ESM, applied as a change to grasslands in HadAM3P and CESM, and ignored in MIROC), allocation of crop changes to grass PFTs for HadAM3P, the subgrid tiling methodology, and differences in the initial land‐cover distributions. Most of these issues cannot be resolved without requiring considerable modification to the land surface schemes of the respective models; however, where possible, the allocation of crop and pasture changes were applied as consistently as possible for both land‐use scenarios.

**Table 4:** Area for the Major PFT Classes for Each Participating Model Including the Net Area Changed (M km^2^) Relative to the PD Land Cover

	Model	CESM	HadAM3P	MIROC	MPI‐ESM
PD	B/S^a^	54.37	37.38	29.36	55.55
	Forest	55.72	25.30	60.16	33.93
	Grass	32.52	23.96	36.71	10.83
	Crop	16.81	39.12	14.73	15.65
	Pasture				31.14
SSP1	B/S^a^	+0.98	+0.07	−0.29	+0.32
	Forest	+3.88	+5.09	−2.70	+1.66
	Grass	−6.36	−3.39	−1.47	+2.96
	Crop	+3.41	−1.77	+4.40	+2.01
	Pasture				−6.95
SSP2	B/S^a^	−0.21	−1.71	−0.41	−1.28
	Forest	−3.62	−1.70	−4.23	−3.02
	Grass	−0.29	+0.15	−2.52	−0.44
	Crop	+5.20	+3.27	+7.15	+4.76
	Pasture				−0.01

a Bare soil and shrub PFTs combined together.

### 3.2 Impact of Climate Target Versus LUC Scenario

**Figure 3:**
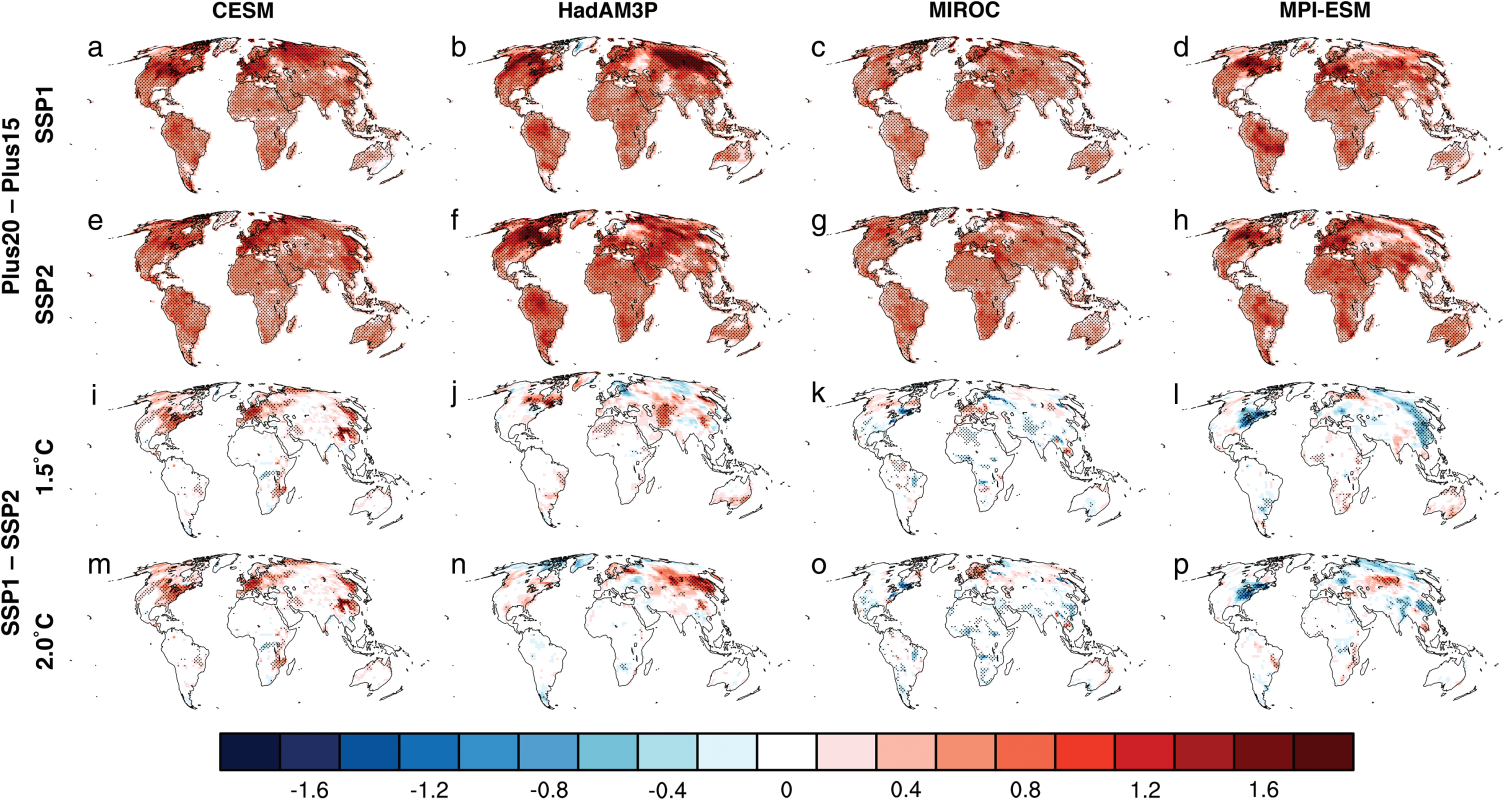
Impact of climate target and land‐use scenario. Mean change in annual maximum daytime 2 m air temperature (TXx, °C) for all models and future scenarios expressed as: PlusZZ_LU1_ minus PlusZZ_LU2_, where ZZ is either 1.5 or 2 and LU is either SSP1 or SSP2. For CESM (a, e, i, and m), HadAM3P (b, f, j, and n), MIROC (c, g, k, and o), and MPI‐ESM (d, h, l, and p). For Plus20_SSP1_ minus Plus15_SSP1_ (a–d), Plus20_SSP2_ minus Plus15_SSP2_ (e–h), Plus15_SSP1_ minus Plus15_SSP2_ (i–l), and Plus20_SSP1_ minus Plus20_SSP2_ (m–p). Stippling denotes where the change is statistically significant at the 95% confidence level (determined from a 1000 bootstrap sampling procedure with a two‐sided test of the paired difference between two means). Oceans are masked in white.

By design of the experiments, the Plus20 experiments yield warmer annual maximum daytime 2 m air temperature (TXx) values than the Plus15 experiments, irrespective of the land‐use scenario (Figures 3a–3h). Indeed, the difference in TXx is often larger than 0.5°C over land, exceeding 1°C in the Northern midlatitude regions for all models. Between the SSPs (Figures 3i–3p), the change in TXx varies across the ESMs. In particular, the MIROC model shows the least sensitivity to the land‐use scenario (Figures 3k and 3o). The CESM model shows the most consistent change in TXx between the SSPs for both climate targets (Figures 3i and 3m). In contrast, the HadAM3P (Figures 3j and 3n) and MPI‐ESM (Figures 3k and 3o) show some sensitivity to the climate target when assessing the change in TXx between the SSPs. For most ESMs, the impact of the SSP scenario on TXx is greatest in the Northern midlatitudes despite significant LUC imposed in Africa, SAM, and Eastern Australia (Figure 2). Furthermore, the change in TXx between the SSPs over NAM, Europe, and parts of Asia is often comparable in magnitude to the change in TXx between the climate targets. Examining the LUC‐induced changes in TXx relative to the present climate decade (Figure S2) also demonstrates sensitivity to both the land‐use scenario and climate target with differences often exceeding 0.5°C. Similar changes in the annual minimum daytime 2 m air temperature (TNn) are also found (Figures S3 and S4), however, the land‐use scenario generally displays a weaker effect on cold extremes. Therefore, our results demonstrate that the land‐use scenarios can have significant impacts on the TXx response to half a degree of warming, albeit with substantial model dependence with respect to the location, sign, and strength of the investigated biogeophysical effect.

### 3.3 LUC Contributions to the Total Temperature Response

**Figure 4:**
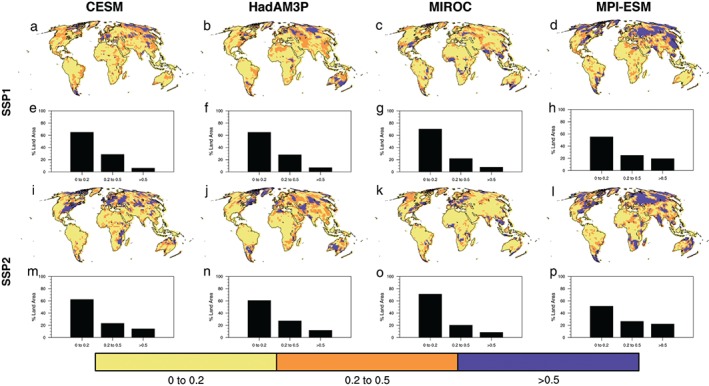
Evaluation of the LUC effect versus total effect of all forcings on the annual maximum daytime 2 m air temperature (TXx) for all models and land‐use scenarios for the Plus15 climate target expressed as: |Plus15_LU_ minus Plus15_Hist_/Plus15_LU_ minus Hist|, where LU is either SSP1 or SSP2. For CESM (a, e, i, and m), HadAM3P (b, f, j, and n), MIROC (c, g, k, and o), and MPI (d, h, l, and p). Panels (a–d) and (i–l) depict the spatial pattern and panels (e–h) and (m–p) depict a histogram of the fraction of land area. For Plus15_SSP1_ minus Plus15_Hist_ (a–h) and Plus15_SSP2_ minus Plus15_Hist_ (i–p). Oceans are masked in white.

In the context of all the forcings applied in the future climate experiments (i.e., Plus15 and Plus20), we examine both the spatial distribution of the LUC contribution to TXx changes (equation (1) in Materials and Methods) and the percentage of land where the change is substantial (Figure 4, shown only for Plus15). Consistent with the results shown in Figure 3, regions where the LUC contribution is greater than 20% of the total TXx response in Plus15 often coincide with the regions where changes in TXx exceed 0.5°C for all models. MPI‐ESM has the largest LUC contribution to the temperature response with ∼20% of the land area in both land‐use scenarios having a >50% change in TXx attributed to the LUC forcing (Figures 4h and 4p). All models show that more than 20% of the change in TXx over at least 20% of the land area can be attributed to the LUC forcing, however, the regions where the LUC forcing has a large impact (explaining >50% of the TXx change) varies between the models. A similar examination of the LUC contribution to changes in TNn (Figure S5) also shows that between 15% and 20% of the land area has a 20–50% change in TNn attributed to LUC for all models. Regions where more than 50% of the TNn response is attributed to LUC are less extensive than for the TXx response and vary between 5% and 15% of the land area. Here, we only show the results for the Plus15 experiments, but note that for the Plus20 experiments, the influence of the land‐use scenario is not as great, due to the larger impact from the other forcings suggesting that the impact of the land‐use scenario decreases in influence for higher emission scenarios. Therefore, LUC has a considerable influence on changes in temperature extremes for low‐emission scenarios.

### 3.4 SEB Decomposition of Temperature Response

**Figure 5:**
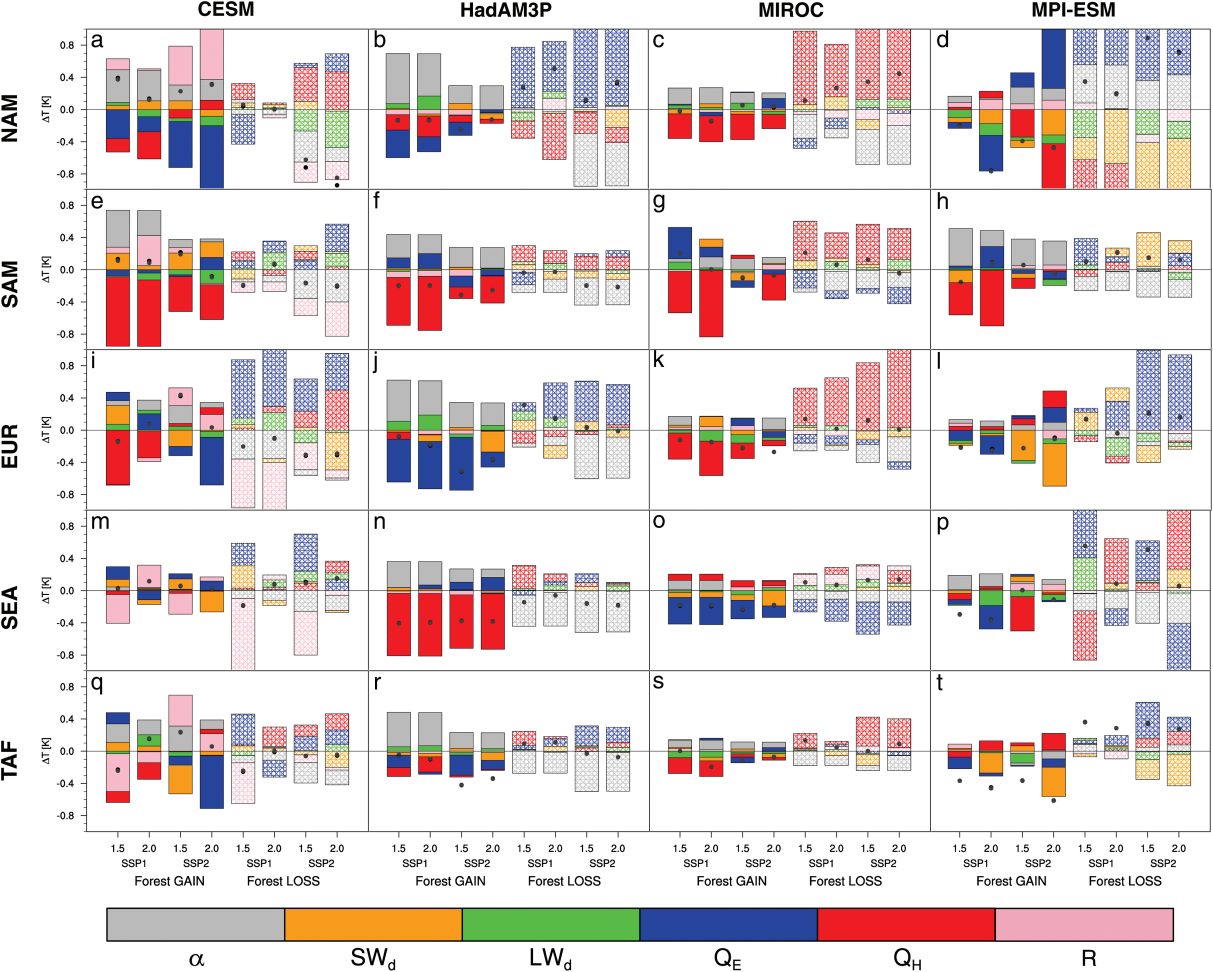
Change in surface temperature as explained by changes in the components of the SEB, corresponding to the conditions when TXx occurs, calculated according to equation (3). Analysis split according to where there is either forest gain or loss, SSP scenario and climate target for different regions: NAM (a–d), SAM (e–h), EUR (i–l), SEA (m–p), and TAF (q–t). For CESM (a, e, i, m, and q), HadAM3P (b, f, j, n, and r), MIROC (c, g, k, o, and s), and MPI‐ESM (d, h, l, p, and t). The black dots denote the actual mean change in surface temperature from the model, the gray dots denote the temperature change derived from the SEB decomposition method, and the histogram denotes the mean change attributed to albedo (α; gray), incoming shortwave radiation (SW_d_; orange), incoming longwave radiation (LW_d_; green), latent heat flux (Q_E_; blue), sensible heat flux (Q_H_; red), and the residual (R; pink). A 5% threshold change in land cover is applied.

We apply the SEB decomposition method (see Section 2) to attribute the TXx response to either the change in available energy (via albedo change) or the partitioning of available energy (through changes in evapotranspiration). Here, we limit the analysis to grid cells where the forest gain or loss exceeds 5% and aggregate the changes in the SEB components for five different regions: NAM, SAM, EUR, SEA, and TAF (Figure 5). The SEB decomposition method is applied using the skin temperature (*T*_s_) rather than the 2 m air temperatures we have used in our previous analysis (Figures 3 and 4). However, the change in *T*_s_ was generally consistent with the TXx changes where they are significant, except for the HadAM3P model (Figures S2 and S6). Generally, there is no consistent SEB response to LUC, neither across the models nor the regions. Furthermore, changes in *T*_s_ are explained by different SEB responses that are quite diverse depending on the type of transition, due to how changes in albedo and evapotranspiration perturb surface climate and the subsequent atmospheric feedback. For example, the HadAM3P model shows *T*_s_ is quite sensitive to sensible heat changes for SAM and SEA where there is an increase in forest extent, however, this sensitivity is not observed when there is forest loss (i.e., one might expect the opposite response for forest gain with a strong positive *Q*_H_ contribution; this is not the case). Furthermore, HadAM3P changes in *T*_s_ seem to be triggered by the change in albedo rather than changes in latent heat which are also considerably smaller in both types of transitions over these regions (Figures 5f and 5n). This in part may also depend on how extensive the LUC was relative to the initial land‐cover extent (Winckler et al., 2017). *T*_s_ in MIROC is also sensitive to sensible heat changes for most regions driven by changes in available energy arising from the albedo change (Figure 5, third column). In contrast for most regions except SAM, MPI‐ESM shows that changes in the partitioning of available energy often have a larger influence on the temperature response than that due to changes in the amount of available energy (Figure 5, fourth column). CESM also shows that the residual term has influence on *T*_s_ that is not evident in the other models (Figure 5, first column). Both CESM and MPI‐ESM show that the albedo change associated with forest extent changes is considerably influential for the temperature response for SAM (Figures 5e and 5h). Generally, Figure 5 demonstrates that the primary driver for the temperature response to LUC is model and region dependent.

### 3.5 Multimodel Spread

**Figure 6:**
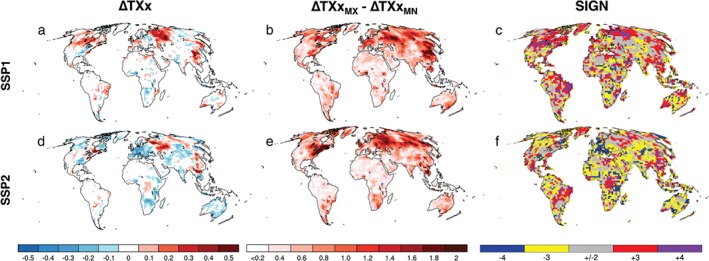
Multimodel response in annual maximum daytime 2 m air temperature (TXx, °C) for the different SSPs for the 1.5°C climate target. For the multimodel mean change (a and d), the multimodel range (b and e), and the agreement on the sign of the TXx change (c and f). For Plus15_SSP1_ minus Plus15_Hist_ (a–c) and Plus15_SSP2_ minus Plus15_Hist_ (d–f). Interpretation for the sign agreement: blue, all models show a temperature decrease; yellow, three models show a temperature decrease; gray regions, no consensus; red, three models show a temperature increase; purple, all models show a temperature increase. Oceans are masked in white.

Aggregating the results across the models for TXx changes relative to the present climate decade indicates that there is a clear dependence on which land‐use scenario is applied (Figure 6). Here, we only show the results for the 1.5°C target as the multimodel aggregates were similar between the climate targets. More specifically, for parts of the Northern midlatitudes, TXx tends to increase under the SSP1 scenario (Figures 6a), while the same regions experience cooler TXx values for the SSP2 scenario (Figures 6d). However, the spread between the models is considerable (also evident from Figure S2) and often exceeding the signal from the multimodel mean (Figures 6b and 6e). This uncertainty is also generally larger over the Northern Hemisphere land. We also examine where the models agree on the sign change and note that in regions where the range is large (e.g., NAM, Europe, and Asia) there is still some consensus on the sign of the change. Similar analysis of the multimodel response for TNn (Figure S7) also indicates that the spread often exceeds the mean signal, however, there is greater consensus on the sign of the change than that for TXx.

## 4 Discussion

We have performed a multimodel experiment (HAPPI‐Land) based on four well‐established independent ESMs to assess the role of LUC in the context of the 1.5 and 2°C climate targets. The results of the HAPPI‐Land multimodel experiment highlight the essential role of LUC in low‐emission scenarios. In particular, the effects of LUC are often regionally larger than 0.5°C and for the Northern midlatitudes, LUC often accounts for more than 20% of the change in temperature extremes for a 1.5°C global mean temperature. The findings, however, also highlight the high level of model discrepancy in the simulated responses with a model spread that often exceeds the mean signal associated with LUC. This is consistent with previous analyses showing important discrepancies in the representation of LUC impacts in the context of historical simulations (Boisier et al., 2012; de Noblet‐Ducoudré et al., 2012; Pitman et al., 2009). The fact that the LUC‐associated uncertainty often exceeds the multimodel mean LUC signal has implications for decision‐makers on what the impacts are for temperature extremes for low‐emission scenarios, as this is both model dependent and land‐use scenario dependent. Indeed, this is a well‐established problem within the climate science community that requires urgent attention to resolve. The upcoming Land‐Use Model Intercomparison Project (LUMIP) intends to examine some of these uncertainties (Lawrence et al., 2016).

We note four important factors that influence the LUC‐based uncertainty. First, the implementation of the socioeconomic pathways and their associated land‐based mitigation activities are based upon assumptions on how future events may evolve that may vary across different IAMs. Here, we only examine two scenarios from one IAM. Considering scenarios from other IAMs that may be based on a different set of assumptions and criteria may lead to different results. Second, the representation of LUC in the respective ESMs differs due to various factors, including vegetation classes, mapping of IAM land use to ESM land use, treatment of pasture, subgrid tiling methodology, and the initial land‐cover distributions. This results in differences in how LUC is reflected by changes in the biogeophysical and biogeochemical characteristics of the land surface. Third, there is the uncertainty on how LUC is related to biogeophysical feedbacks associated with changes in albedo, evapotranspiration, or roughness length that depend on the type of transition and location that it occurs. Finally, even if the challenges in LUC implementation could be resolved between the land surface components of the ESMs, differences in the response to LUC may still exist due to uncertainty on the atmospheric feedback (Hirsch et al., 2015). Therefore, the uncertainty arising from the implementation of LUC within ESMs presents a significant challenge to the climate science community that requires a resolution. In particular, if these models are used to evaluate local to regional scale impacts associated with LUC and climate change then efforts to reduce uncertainty are critical.

## 5 Conclusions

The first results from the four ESMs participating in HAPPI‐Land demonstrate that for low‐emission scenarios compatible with a global mean warming of 1.5°C and 2°C, the land‐use scenario can contribute to substantially different outcomes. We also examine the potential role for land use to introduce uncertainty in predicting extremes for half a degree of warming and find that consensus is lacking between ESMs due to the considerable uncertainty on how the biogeophysical feedbacks of LUC influence climate extremes in addition to the different LUC representations between ESMs. As demonstrated in this study, LUC is an important component of low‐emission scenarios, accounting for more than 20% of the change in temperature extremes for large land areas concentrated over the Northern Hemisphere. Therefore, our results highlight the urgent need to better understand the biogeophysical impacts of LUC to improve understanding of how they contribute to regional changes in extremes. This is critical for enabling their implementation in IAMs and ultimately contribute to the design of more sustainable development pathways.

## Supporting information

Supporting Information:
Figure S1.
Figure S2.
Figure S3.
Figure S4.
Figure S5.
Figure S6.
Figure S7.
