# Supplementary material for: Biogeophysical Impacts of Land‐Use Change on Climate Extremes in Low‐Emission Scenarios: Results From HAPPI‐Land
Source: Earths Future. 2018 Mar 8;6(3):396–409. doi: 10.1002/2017EF000744 (PMC5993232; doi:10.1002/2017EF000744)
Supplement: Supplementary file 1 — Supporting Information: Figure S1. Figure S2. Figure S3. Figure S4. Figure S5. Figure S6. Figure S7. [file EFT2-6-396-s001.pdf]

AGU PUBLICATIONS

Earth's Future

Supporting Information for

Biogeophysical impacts of land use change on climate extremes in low emission scenarios: Results from HAPPI-Land

A. L. Hirsch[1], B. P. Guillod[1,2], S. I. Seneviratne[1], U. Beyerle[1], L. R. Boysen[3], V. Brovkin[3], E. L. Davin[1], J. C. Doelman[4], H. Kim[5], D. M. Mitchell[6], T. Nitta[5], H. Shiogama[7], S. Sparrow[8], E. Stehfest[4], D. P. van Vuuren[4,9], S. Wilson[10,11]

[1]*Institute for Atmospheric and Climate Science, Eidgenössische Technische Hochschule (ETH) Zurich, 8092 Zurich, Switzerland*

[2]*Institute for Environmental Decisions, Eidgenössische Technische Hochschule (ETH) Zurich, 8092 Zurich, Switzerland*

[3]*Max Planck Institute for Meteorology, 20146 Hamburg, Germany*

[4]*PBL Netherlands Environmental Assessment Agency, 2594 AV Den Haag, the Netherlands*

[5]*Institute of Industrial Science, the University of Tokyo, 153-8505 Tokyo, Japan*

[6]*School of Geographical Sciences, University of Bristol, BS8 1SS Bristol, UK*

[7]*Center for Global Environmental Research, National Institute for Environmental Studies, 16-2 Onogawa, Tsukuba, Ibaraki 305-8506, Japan*

[8]*Oxford e-Research Centre (OeRC), University of Oxford, OX1 3QG Oxford, UK*

[9]*Copernicus Institute for Sustainable Development, Utrecht University, 3584 CS Utrecht, The Netherlands*

[10]*Met Office Hadley Centre, EX1 3PB Exeter, UK*

[11]*NCAS-CMS, Department of Meteorology, University of Reading, RG6 6AH Reading, UK*

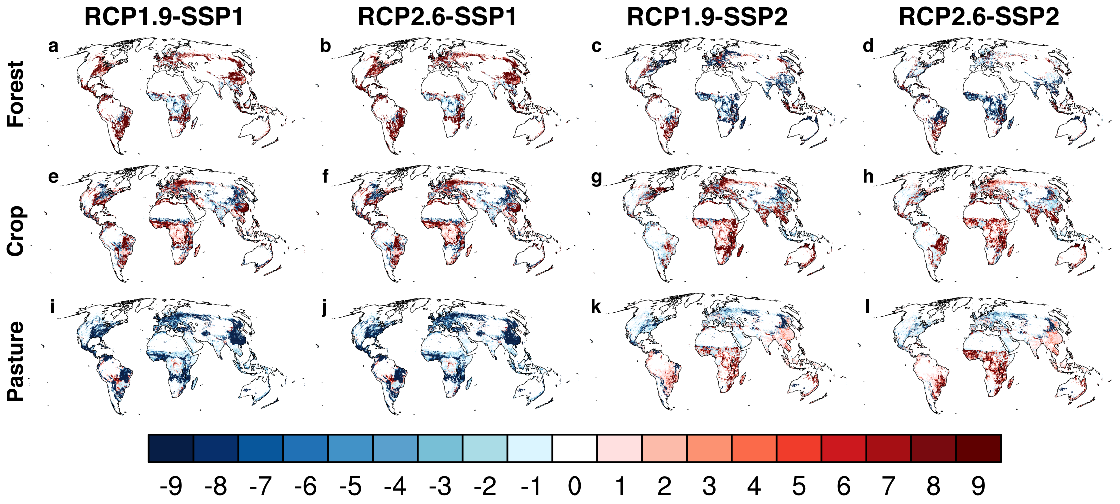

Figure S1: Percent fraction change (2100 minus 2010) in main land cover types taken from four different IMAGE land use scenarios. For forests (a-d), cropland (e-h), and pasture (i-l) for the scenarios RCP1.9-SSP1 (a, e, and i), RCP2.6-SSP1 (b, f, and j), RCP1.9-SSP2 (c, g, and k), and RCP2.6-SSP2 (d, h, and l).

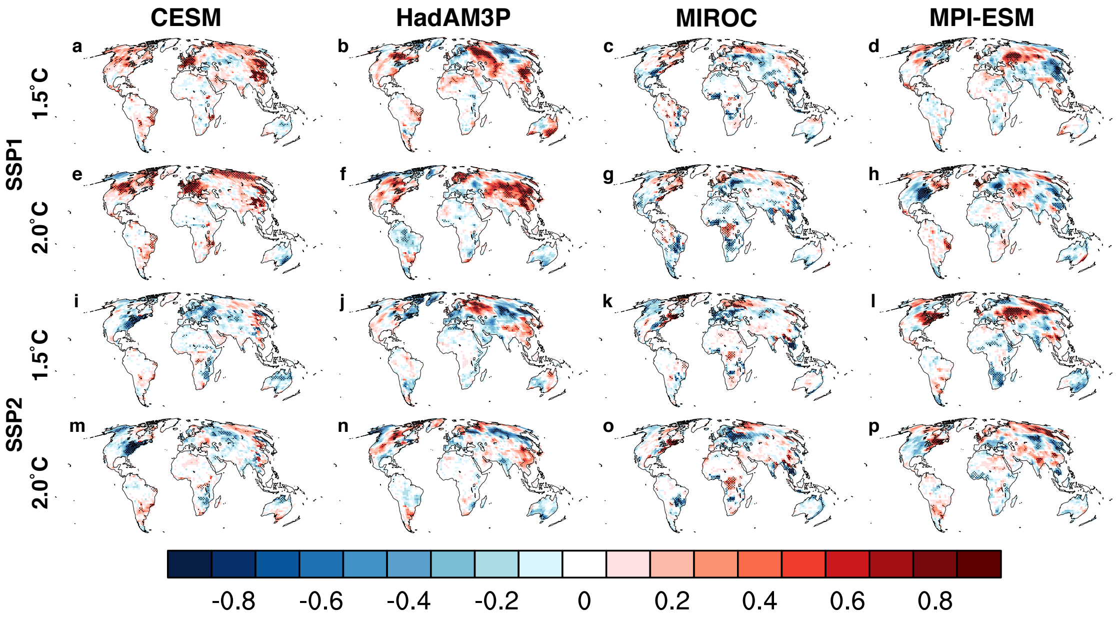

Figure S2: Mean change in annual maximum daytime 2m air temperature [TXx; ˚C] for all models and future scenarios expressed as: $PlusZZ_{LU}$ minus $PlusZZ_{Hist}$ where ZZ is either 1.5 or 2 and LU is either SSP1 or SSP2. For CESM (a, e, i, and m), HadAM3P (b, f, j, and n), MIROC (c, g, k, and o), and MPI-ESM (d, h, l, and p). For $Plus15_{SSP1}$ minus $Plus15_{Hist}$ (a-d), $Plus20_{SSP1}$ minus $Plus20_{Hist}$ (e-h), $Plus15_{SSP2}$ minus $Plus15_{Hist}$ (i-l), and $Plus20_{SSP2}$ minus $Plus20_{Hist}$ (m-p). Note that stippling denotes where the change is statistically significant at the 95% confidence level (determined from a 1000 bootstrap sampling procedure with a two-sided test of the paired difference between two means). Note that oceans are masked in white.

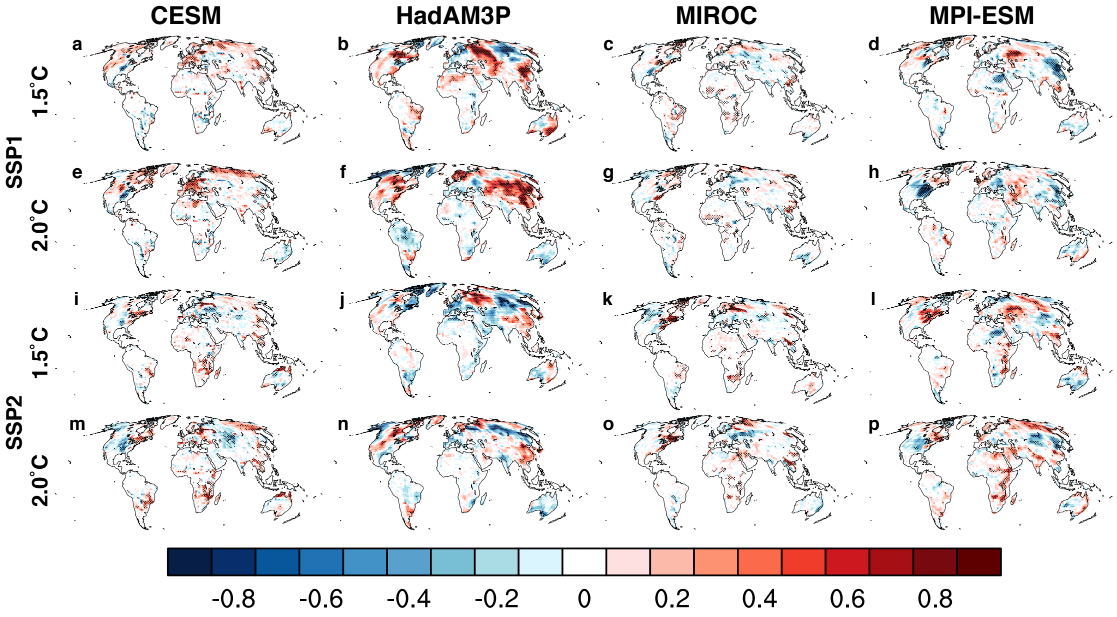

Figure S3: As in Figure S2 but for the annual minimum night-time 2m air temperature [TNn; ˚C].

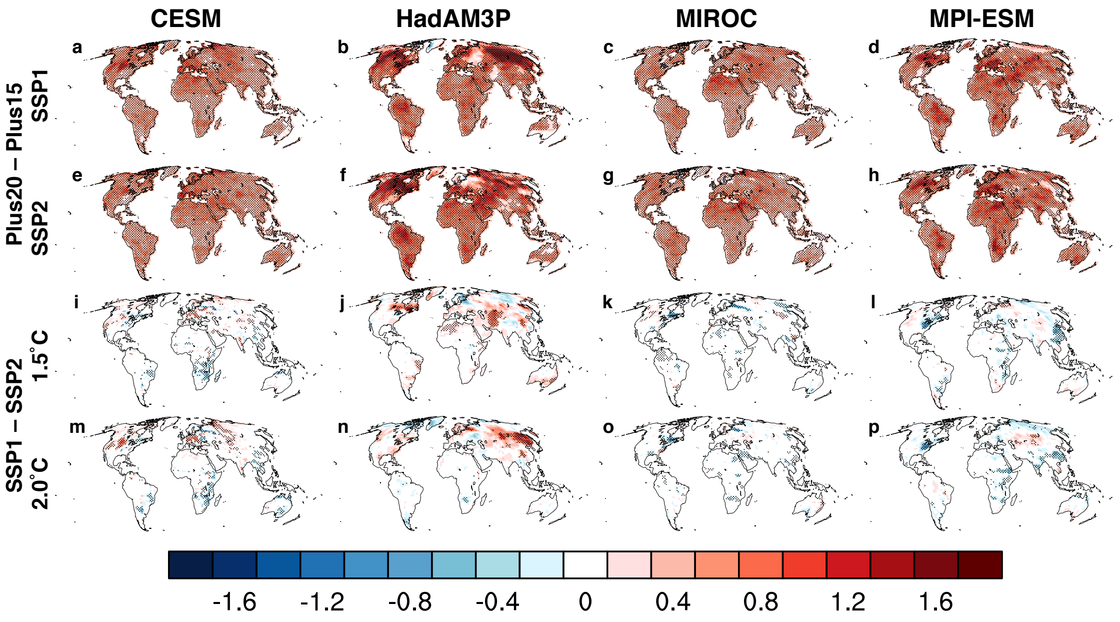

Figure S4: Impact of climate target and land use scenario. Mean change in annual minimum night-time temperature [TNn; ˚C] for all models and future scenarios expressed as: $PlusZZ_{LU1}$ minus $PlusZZ_{LU2}$ where ZZ is either 1.5 or 2 and LU is either SSP1 or SSP2. For CESM (a, e, i, and m), HadAM3P (b, f, j, and n), MIROC (c, g, k, and o), and MPI-ESM (d, h, l, and p). For $Plus20_{SSP1}$ minus $Plus15_{SSP1}$ (a-d), $Plus20_{SSP2}$ minus $Plus15_{SSP2}$ (e-h), $Plus15_{SSP1}$ minus $Plus15_{SSP2}$ (i-l), and $Plus20_{SSP1}$ minus $Plus20_{SSP2}$ (m-p). Note that stippling denotes where the change is statistically significant at the 95% confidence level (determined from a 1000 bootstrap sampling procedure with a two-sided test of the paired difference between two means). Note that oceans are masked in white.

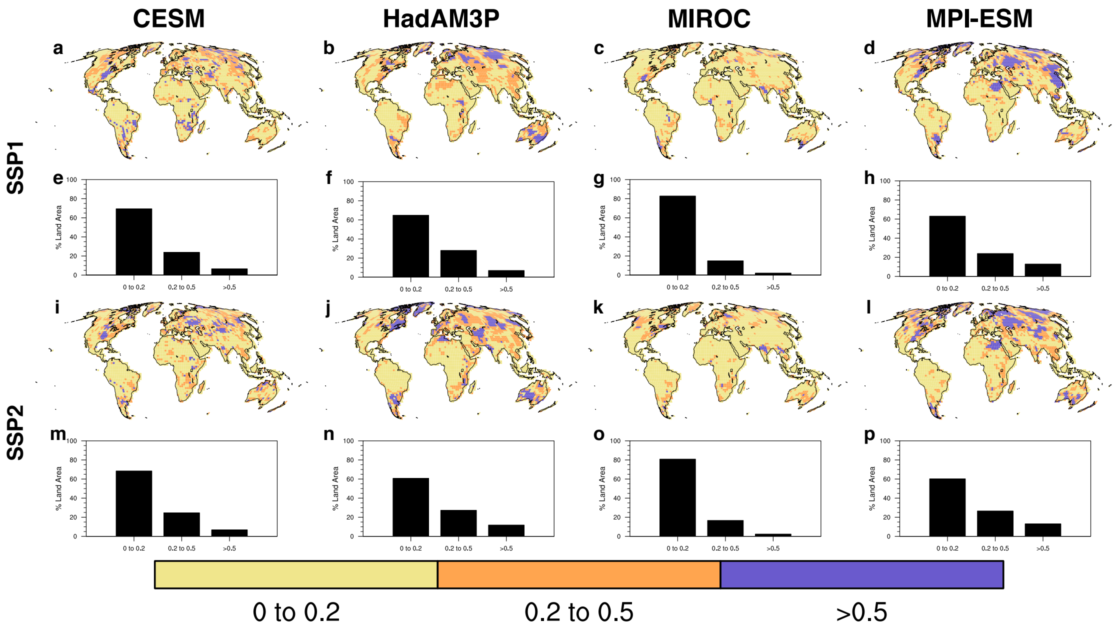

Figure S5: Evaluation of the LUC effect vs. total effect of all forcings on TNn for all models and land use scenarios for the Plus15 climate target expressed as: | $Plus15_{LU}$ minus $Plus15_{Hist}$ / $Plus15_{LU}$ minus Hist | where LU is either SSP1 or SSP2. For CESM (a, e, i, and m), HadAM3P (b, f, j, and n), MIROC (c, g, k, and o), and MPI-ESM (d, h, l, and p). Panels (a-d) and (i-l) depict the spatial pattern and panels (e-h) and (m-p) depict a histogram of the fraction of land grid cells of this diagnostic. For $Plus15_{SSP1}$ minus $Plus15_{Hist}$ (a-h), and $Plus15_{SSP2}$ minus $Plus15_{Hist}$ (i-p). Note that oceans are masked in white.

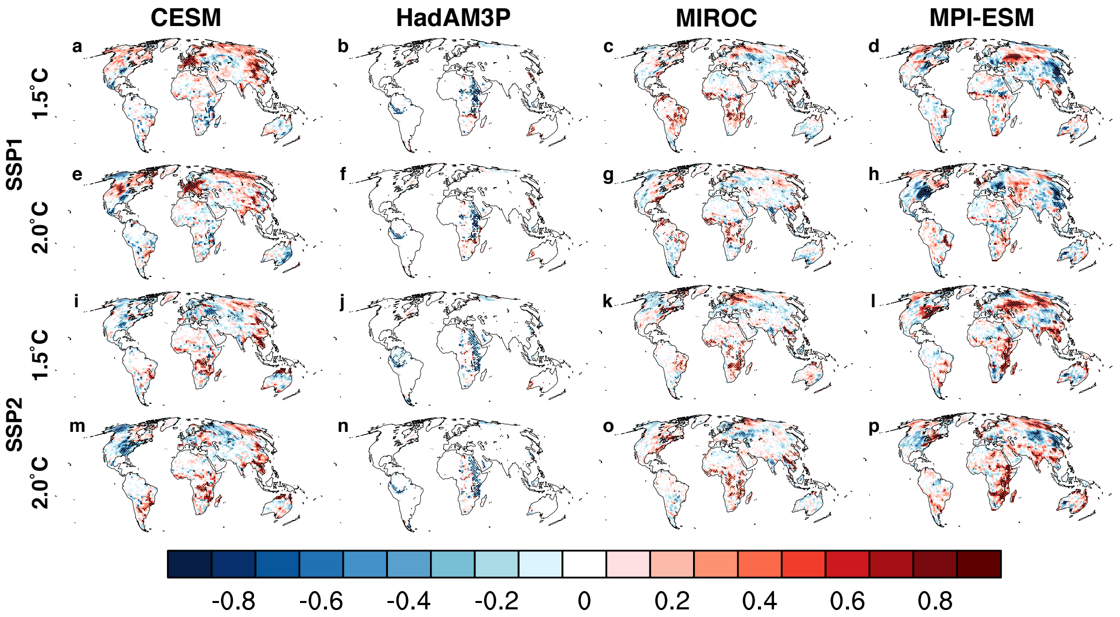

Figure S6: As in Figure S2 but for the surface temperature corresponding to the day that TXx occurs [T_s; ˚C].

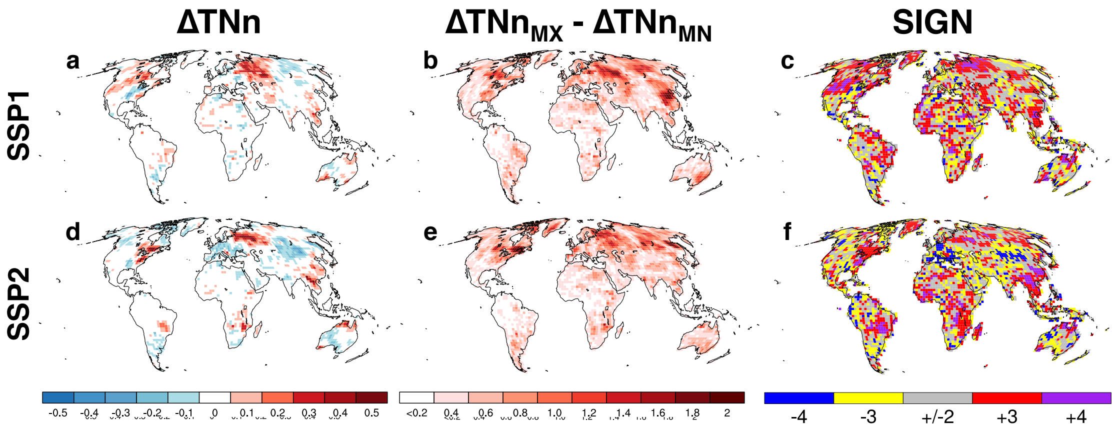

Figure S7: Multi-model response in annual minimum night-time 2m air temperature [TNn; ˚C] for the different SSPs for the 1.5˚C climate target. For the multi-model mean change (a and d), the multi-model range (b and e) and the agreement on the sign of the TNn change (c and f). For $Plus15_{SSP1}$ minus $Plus15_{Hist}$ (a-c) and $Plus15_{SSP2}$ minus $Plus15_{Hist}$ (d-f). Interpretation for the sign agreement: blue = all models show a temperature decrease, yellow = 3 models show a temperature decrease, grey regions = no consensus, red = 3 models show a temperature increase, and purple = all models show a temperature increase. Note that oceans are masked in white.
